# Randomized double blind clinical trial on the effect of oral α-cyclodextrin on serum lipids

**DOI:** 10.1186/s12944-016-0284-6

**Published:** 2016-07-12

**Authors:** Marcelo J. A. Amar, Maryann Kaler, Amber B. Courville, Robert Shamburek, Maureen Sampson, Alan T. Remaley

**Affiliations:** Lipoprotein Metabolism Section, Cardio-Pulmonary Branch, National Heart, Lung, and Blood Institute, National Institutes of Health, Building 10, Room 8 N-228, 10 Center Drive MSC 1666, Bethesda, MD USA

**Keywords:** α-cyclodextrin, TC, Total cholesterol, TG, Triglycerides, LIRI, Lipoprotein Insulin Resistance Index, LDL-C, Low density lipoprotein cholesterol, HDL-C, High density lipoprotein cholesterol, BAS, Bile acid sequestrants

## Abstract

**Background:**

This single center, double-blinded, cross-over, placebo controlled clinical trial investigated the effect of oral α-cyclodextrin (α-CD), a soluble dietary fiber, on blood lipid and lipoprotein levels in healthy human subjects. α-CD, a cyclical polymer containing 6 glucose subunits, is currently sold as an over the counter food supplement and is also a common additive in many foods. α-CD forms a hydrophobic central cavity that binds lipids and has been shown in animal studies and in previous clinical trials to alter plasma lipid levels.

**Methods:**

We screened for healthy subjects, males and females, between ages 18 to 75. Out of total 103 subjects interviewed, 75 subjects completed the study. Qualified individuals in each gender group were randomized into two groups in terms of which treatment arm they received first (placebo vs. α-CD, receiving 6 grams P.O. a day, for 12–14 weeks with a 7 day wash out between arms). The primary outcome variable, plasma total cholesterol, as well as other tests related to lipids and lipoprotein and glucose metabolism, were measured at baseline and at the end of each arm of the study.

**Results:**

α-CD was well tolerated; no serious adverse events related to α-CD were observed. Approximately 8 % of the subjects on α-CD complained of minor gastrointestinal symptoms versus 3 % on placebo (*p* = 0.2). Small-LDL particle number decreased 10 % (*p* < 0.045) for subjects on α-CD versus placebo. Fasting plasma glucose (1.6 %, *p* < 0.05) and Insulin resistance index (11 %, *p* < 0.04) were also decreased when on α-CD versus placebo.

**Conclusion:**

α-CD treatment appears to be safe and well tolerated in healthy individuals and showed a modest reduction in small LDL particles, and an improvement in glucose related parameters.

**Trial registration:**

NCT01131299

## Background

Despite numerous therapeutic advances, CVD remains the leading cause of morbidity and mortality in developed countries [1]. The major modifiable risk factors for CVD include elevated low density lipoprotein cholesterol (LDL-C), decreased high density lipoprotein cholesterol (HDL-C), diabetes, cigarette smoking, inactivity, obesity and a poor diet, which is often low in soluble fiber and high in saturated and trans fats [2]. Although statins are the most effective therapeutic agents for reducing CVD risk, they only reduce cardiovascular events by approximately 30 % [3]. Soluble dietary fibers and bile acid sequestrants are two other currently used agents for lowering serum lipids [4]. Bile acid sequestrants (BAS) are among the oldest lipid-altering drugs and have been known for decades to also improve glucose control and to reduce CVD risk [5]. Both of these agents, however, have relatively poor compliance because of the large amount of agent that is needed to achieve a lipid lowering effect, poor palatability and gastrointestinal discomfort [6].

Soluble dietary fibers, which exist in a normal diet and are commonly used food additives, are also known to reduce blood cholesterol levels [7]. A recent meta-analysis, however, concluded that most soluble fibers reduce total cholesterol (TC) by relatively small amounts, approximately 1.6 mg/dL per gram of soluble fiber [8]. For a normal-weight subject following a recommended diet of 2000 kcal/day containing 30 % fat, this amounts to only about a 4.6 % reduction in total cholesterol (TC) levels. Another meta-analysis of 8 controlled intervention trials reported only a 4 % reduction in TC levels in hypercholesterolemic subjects that consumed as much as 10 g psyllium per day [9].

α-CD is a soluble fiber derived from corn and is used as an ingredient in many foods, such as bread rolls, crackers, juices, chewing gum and reduced fat spreads [10]. Based on safety data, α-CD has been granted Generally Recognized As Safe (GRAS) status by the FDA [11, 12]. It is commonly added to food as a fiber supplement but is also used as a stabilizer of flavors, colors, vitamins and fatty acids and for improving the mouth-feel of beverages [11]. It is also sold for human consumption as a dietary food supplement and is distributed by many health food stores. Unlike most other soluble fibers, which are long linear or branched polymers, α-CD is a cyclic polymer and forms a toroidal-like structure. It contains 6 glucose molecules, which form a central hydrophobic cavity with high affinity for lipids, such as cholesterol and other fats [11]. One possible advantage of α-CD over other soluble fibers or over BAS in lowering plasma lipids is that less agent may be required to complex fat in the diet, because of its relative high affinity for lipid. One gram of α-CD has been shown to bind as much as nine grams of dietary fat [13]. It is also tasteless and anecdotal reports from subjects taking α-CD as a dietary food supplement suggest that it is well tolerated and causes minimal gastrointestinal discomfort.

Our previous animal studies, in mice, demonstrated that the addition of oral α-CD to regular chow diet improved the lipid profile by lowering pro-atherogenic lipoproteins and trans-fatty acids and by decreasing the ratio of saturated and trans-fatty acids to polyunsaturated fatty acids [14]. In this study, low-density lipoprotein receptor knockout mice were fed a “Western diet” (21 % milk fat) with or without 2.1 % of α-CD (10 % of dietary fat content) for 14 weeks. At sacrifice, there was no difference in body weight; however, significant decreases were observed in plasma cholesterol (−15.3 %), free cholesterol (−20 %), cholesteryl esters (−14 %), and phospholipids (−17.5 %) levels in mice treated with α-CD compared with control mice. Furthermore, α-CD improved the blood fatty acid profile, reducing the saturated fatty acids (−4.5 %) and trans-isomers (−11 %), while increasing unsaturated fatty acids (2.5 %) [15]. Results from this study are consistent with other animal studies, showing the possible utility of α-CD as a dietary supplement for decreasing serum lipids [12, 16, 17].

There have only been, however, a limited number of human studies on α-CD [13, 18, 19]. In one study conducted by Grunberger and colleagues in obese (BMI > 30) type II diabetic patients, treatment with α-CD (6 g a day) for 3 months led to a reduction or maintenance of body weight, an increase in insulin sensitivity as a result of increased levels of adiponectin, and a lowering of plasma triglyceride and LDL cholesterol, with no apparent side effects [13]. A second study conducted by Buckley and colleagues also on diabetic patients aimed to understand the effects of α-CD on acute glucose and insulin response to a standard carbohydrate meal (50 g of carbohydrate in white rice) containing 0 to 10 g of α-CD. They observed significantly reduced post-prandial plasma glucose levels without any increase insulin response for subjects ingesting α-CD. It was also observed that α-CD was associated with an increased incidence of minor gastrointestinal complaints (stomach ache, nausea, bloating), but this was primarily observed in subjects on a low fat diet [19].

In the present study, we examined in a randomized cross-over placebo-control design the effect of oral α-CD in a relatively healthy control population without diabetes and or obesity to determine if α-CD supplementation could have broader health benefits in the general population. The primary outcome variable was plasma total cholesterol but other measures related to lipid and glucose control were also assessed.

## Methods

### Subject and experimental participants

**Fig. 1:**
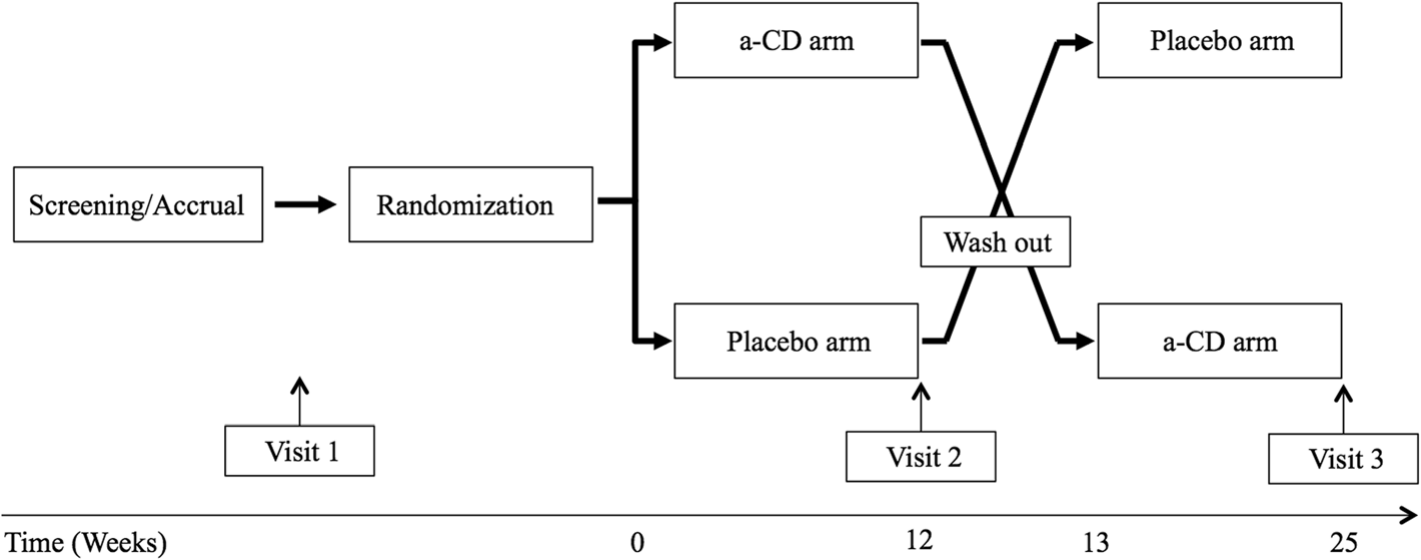
Experimental design of the study

We screened 103 subjects, males and females, between the ages of 18–75. 75 subjects completed the study, and were randomized into two groups first receiving either placebo or α-CD followed by the other treatment (Fig. 1).

The inclusion and exclusion criteria were designed to recruit a relatively healthy population and subjects with diabetes were specifically excluded (Table 1). The study was approved by the NHLBI IRB under protocol # 10-H-0088 and FDA IND # 108017. All participants signed a consent form. Out of the 103 subjects enrolled in the study, 75 completed the study. Fifteen subjects were excluded for laboratory abnormalities at baseline and 13 subjects were withdrawn prior to completion of the study for non-compliance with the drug intake schedule.

**Table 1:** Inclusion and exclusion criteria of the study

Inclusion criteria
• Males and females, ages of 18–75.
• Understands and provides written, informed consent.
Exclusion Criteria
• Pregnancy or breastfeeding.
• BMI < 18.5
• Weight (change > 10 % over in the past 3 months).
• Low-fat (<20 %) diet.
• Less than 3 meals/snacks per day
• Use of medications for at least six weeks: soluble fiber supplements, BAS, plant sterol supplements, long term antibiotics, anticoagulants, anticonvulsants, antiarrhythmics, cyclosporine, mycophenolate, synthroid, vitamin A, E and K or drugs taken with a meal.
• Chronic diarrhea, gastric bypass or lapband procedures, ostomies, bowel motility problems, or conditions that could affect intestinal fat absorption.
• New or multiple medications.
• Type I or type II diabetes.
• Use of α-CD in any of its commercial form.
• Condition or disorder that may affect the outcome of the study or the safety of the volunteer.

### Study design

This was a single center, double-blinded, cross-over, placebo-controlled trial (Fig. 1). The individuals in each gender group, who qualified for the study, were randomized into two groups in terms of which treatment arm they received first (placebo or α-CD). There were a total of 3 visits for this study. Eligible subjects received placebo pills or α-CD (6 grams P.O. a day; two 1 gram tablets per meal) for 12–14 weeks, with a one week of wash out period between arms (Fig. 1). To control for diet and exercise changes, a seven day food diary was obtained prior to the initiation of each study arm and were analyzed, using Nutrition Data Systems for Research, versions 2010–2013 (Mineapolis, MN). In addition, a member of the research team periodically contacted the subjects to monitor compliance. A short physical activity assessment and gastrointestinal symptoms were recorded during each visit.

α-CD was manufactured by Wacker Biochem (Adrian, MI), under GMP guidelines. The pills containing 1000 mg of α-CD plus Croscarmelose Sodium, Stearic Acid, Magnesium Stearate, Silicon Dioxide as excipients were purchased from ArtJen Complexus Holdings Corp. Identical looking placebo pills were also purchased from ArtJen Complexus Holdings Corp. Placebo pills contained 300 mg of Calcium from the Di-Calcium phosphate, 160 mg of cellulose per tablet and same excipients as α-CD pills.

### Sample analysis

Approximately 40 mL of fasting blood samples were collected at each visit and used to perform the following routine laboratory tests: Sodium (Na), Potassium (K), Chloride (Cl), Bicarbonate, Creatinine, Glucose, Urea nitrogen (BUN), Albumin, Calcium, Alkaline Phosphatase, ALT/GPT, AST/GOT, Total Bilirubin, Total Protein, Lipid Panel, Lipoprotein NMR profile, Thyroid Panel, hs-CRP, vitamin A, vitamin E, carotene, HbA1C, and insulin. These tests were performed in the Department of Laboratory Medicine in the NIH Clinical Center (CC).

### Statistical analysis

It was estimated that a minimum sample size of approximately 62 subjects was needed to detect an 8 mg/dl change in total plasma cholesterol levels between the placebo and α − CD treatment periods, with an 80 % power, by using the normal approximation to a one-sample (paired), two-sided *t*-test at an alpha = 0.05. A standard deviation of 20 mg/dl was assumed for the paired differences in plasma cholesterol levels between the two treatment periods. An expected attrition rate was set to 30 %, therefore, the power calculations predicted that a total sample size of *n* = 70 was needed for the study.

Descriptive statistics were calculated for all variables. All response variables were assessed for conformance to the normal distribution and transformed as needed to meet the assumptions of normal distribution and homogeneity between periods. Statistically significant differences in response variables for subjects on placebo versus α-CD treatment arms were determined by two-tailed paired *t*-test analysis. Chi-square analysis was used to compare the frequency of side effects on placebo versus the α-CD treatment. HOMA index was calculated as previously described [20]. All statistical analysis were done with JMP by SAS Institute and Graphpad Prism v. 6.0

## Results

### Baseline patient characteristics

A total of 103 subjects were screened and 89 subjects who met the inclusion and exclusion criteria were enrolled into the study. 2 subjects were later excluded during the study for non-compliance and 12 subjects voluntarily withdrew after starting the study, leaving a total of 75 subjects that completed the study (34 males and 41 females). Of the 12 subjects that withdrew while on the study, one subject withdrew before starting the treatment, and 11 subjects withdrew while taking the study medication; 8 were on the α-CD arm (4 because of gastrointestinal symptoms), and 3 were on the placebo arm (2 because of gastrointestinal symptoms; *p* = NS). No other common cause for withdrawing from the study was given.

Baseline characteristics for the subjects recruited for the study are shown in Table 2. The mean age of the participants were 34 ± 12 years and they had a mean BMI of 25 ± 4 kg/m2. The subjects were mainly of white (71.8 %); 12.6 % were of Asian/Pacific Islander descent and 9.7 % were African Americans.

**Table 2:** Baseline anthropometric characteristics of subjects recruited

Age (years)	n	%
18 − 21	2	1.9
22 − 30	51	49.5
31 − 40	25	24.3
41 − 50	10	9.7
51 − 60	10	9.7
61 − 65	3	2.9
≥66	2	1.9
	Mean	SD
Age (years)	34	12.4
Sex	n	%
Male	43	41.7
Female	60	58.3
Ethnicity	n	%
White	74	71.8
Black	10	9.7
Asian or Pacific Islander	13	12.6
Hispanic	2	1.9
American Indian or Alaskan	0	0.0
Unknown	4	3.9
Anthropomorphic measures	Mean	SD
BMI (Kg/m^2^)	25	3.9
Weight (Kg)	75	17
Systolic Blood Pressure (mmHg)	119	12
Diastolic Blood Pressure (mmHg)	73	10

(*n* = number of subjects; % = percentage of total subjects recruited)

The healthy subjects were recruited from the Bethesda, MD area and generally presented with relatively normal clinical laboratory test values, including those related to serum lipids and lipoproteins (Table 3).

**Table 3:** Lipid profiles and laboratory values of subjects in the placebo and α-CD groups at baseline

Baseline values / reference range	Mean	SEM
Safety parameters		
CRP HS (<3.0 mg/L)	1.4	0.2
AST (9–34 U/L)	19.9	0.1
ALT (6–41 U/L)	29.3	1.5
TSH (0.40 − 4.00 mcIU/mL)	1.6	0.1
Urea (8–22 mg/dL)	3.6	0.5
Creatinine (0.56 − 1.16 mg/dL)	0.8	0.1
Albumin (3.5 − 5.2 g/dL)	4.2	0.1
Alk Phosp (35–105 U/L)	57.8	1.9
Vitamin A (24–85 mcg/dL)	55.5	1.6
Vitamin D (18–78 pg/mL)	54.5	1.7
Vitamin E (5.0 − 19.0 mg/L)	10.9	0.5
RBC (3.93 − 5.22 M/uL)	4.7	0.1
WBC (3.98 − 10.04 K/uL)	5.4	0.1
Lipids and Lipoproteins		
Cholesterol (<200 mg/dL)	169.0	4.1
Triglycerides (<150 mg/dL)	94.0	5.9
LDL-C (<100 mg/dL)	101.0	4.0
HDL-C (>40 mg/dL)	59.6	2.0
Glucose metabolism		
Glucose (74–106 mg/dL)	86.4	0.8
Hgb A1C (4.0 − 6.0 %)	5.3	0.1
Insulin (2.6 − 24.9 mcU/mL)	6.6	0.5
LIRI^a^ (%, by NMR)	1.4	0.1
HOMA IR index	1.4	0.1

^a^Lipoprotein Insulin Resistance Index [25]

### Diet and exercise levels

Seven days before each visit, a food diary and an exercise log was recorded by each subject and reviewed during the nutritional assessment part of each visit.

The average energy and micronutrients intake during the study is displayed in Table 4. No seasonal change in food composition was observed during the study. Overall, the mean dietary nutrient intakes at baseline were also similar in crossover groups. No significant change in exercise levels or food consumption were observed during the study.

**Table 4:** Average Daily Dietary Intake

Nutrient/food group	Mean	SD
Energy (Kcal/d)	2214.6	623.4
Protein (% energy)	17.3	4.1
Fat (% energy)	33.6	5.5
Carbohydrate (% energy)	46.6	8
Alcohol (% energy)	2.7	3.2
Grain Servings^a^	6.9	2
Fruit Servings^a^	1.9	1.5
Vegetable Servings^a^	3.7	1.6
Protein Servings^a^	6.6	2.9
Dairy Servings^a^	2.1	1.1

Macronutrient composition of diet consumed by subjects and monitored by 7 days food records as described in the methods section

^a^Servings were normalized for 2000 kcals/d

### Safety and tolerability

Besides the main outcome parameters related to lipids, a panel of general laboratory tests was also monitored to assess the safety of α-CD treatment (Table 5). No significant differences were observed for any of the safety tests for when patients were on placebo versus α-CD, including those related to fat malabsorption (Vitamin A, D, and E).

**Table 5:** Changes in safety values of subjects after 12–14 weeks on placebo or α-CD either measured by conventional biochemistry methods or by NMR (mean ± SEM)

Safety parameters	Placebo	α-CD	*p*
CRP HS (<3.0 mg/L)	2.1 ± 0.5	2.0 ± 0.4	0.8
AST (9–34 U/L)	20.6 ± 0.7	19.2 ± 1.1	0.1
ALT (6–41 U/L)	28.6 ± 1.4	26.9 ± 1.1	0.08
TSH (0.40 − 4.00 mcIU/mL)	1.9 ± 0.1	1.9 ± 0.1	0.7
Urea (8–22 mg/dL)	13.3 ± 0.5	12.9 ± 0.5	0.4
Creatinine (0.56 − 1.16 mg/dL)	0.8 ± 0.2	0.8 ± 0.2	0.8
Albumin (3.5 − 5.2 g/dL)	4.2 ± 0.1	4.2 ± 0.1	0.8
Alk Phosp (35–105 U/L)	57.5 ± 1.8	57.5 ± 1.7	0.9
Vitamin A (24–85 mcg/dL)	55.9 ± 1.6	55.4 ± 1.6	0.6
Vitamin D (18–78 pg/mL)	52.3 ± 1.6	53.9 ± 1.9	0.3
Vitamin E (5.0 − 19.0 mg/L)	10.8 ± 0.4	10.8 ± 0.3	0.9
RBC (3.93 − 5.22 M/uL)	4.6 ± 0.1	4.6 ± 0.1	0.4
WBC (3.98 − 10.04 K/uL)	5.5 ± 0.1	5.4 ± 0.1	0.4

Overall, the α-CD treatment (6 grams a day) was reasonably well tolerated. There were a total of 35 adverse events (A.E.) and 2 serious adverse events (S.A.E.) as listed in Table 6. The two serious adverse events were considered to be unrelated to the treatment (emergency appendectomy, while on placebo and enrollment in another conflicting clinical trial). A side effect possibly related to the intake of α-CD was mild GI symptoms, which were more frequent on the α-CD arm (8 %) versus the placebo arm (3 %), but this did not reach statistical significance (*p* = 0.19). Four subjects, however, discontinued the study while on α-CD because of GI complaints. Two subjects discontinued the study because of GI complaints while on placebo (*p* = NS).

**Table 6:** Adverse events observed during the study

Adverse Events			Probably/possible related AE		
A.E. (Total *n* = 35)	n	%		Study arm^a^	
Expected AE	10	28.6		α-CD	Placebo
Probably related AE	9	25.7	Abdominal Pain	1	1
Possibly related AE	2	5.7	Intestinal Gas	3	0
Unrelated	14	40.0	Nausea	1	0
			Diarrhea	2	1
S.A.E (Total *n* = 2)	n	%	Urinary Urgency	0	1
Expected SAE	0	0	Dyspepsia	2	0
Related SAE	0	0	Cramps	1	0
Unrelated	2	100	Increased frequency	1	0

A.E. and S.A.E. were classified as expected/unexpected. Unexpected A.E. or S.A.E. were classified as related, probably, possible or unrelated to the treatment

^a^Chi-square *p* value = 0.19

### Changes in lipid parameters

The results related to the main lipid and lipoprotien outcome parameters are shown in Table 7. No statistially signficant differences were observed in total cholesterol or the other commonly measured lipid and lipoprotein test parmeters for when patients were on α-CD treatment versus placebo. Similarly, there was no change in the LDL particle number (LDL-p) as determined by NMR analysis [21]. The size distribution of LDL subractions, however, were different on α-CD treatment versus placebo. After 12–14 weeks on α-CD treatment, there was a 10 % reduction (*p* < 0.045) in small LDL-p compared to placebo (Table 7). In numerous studies, it has been shown that small LDL is more proatherogenic than larger LDL subfractions [22–24]. The mean age, BMI or ethnic distribution did not statistically differ between the responders with a reduction of small LDL-p on α-CD and the non-responders.

**Table 7:** Changes in Lipid, apolipoprotein and lipoprotein values of subjects after 12–14 weeks on placebo or α-CD either measured by conventional biochemistry methods or by NMR (mean ± SEM)

Lipids and Lipoproteins	Placebo	α-CD	*p*
Cholesterol (<200 mg/dL)	180 ± 4	180 ± 4	0.82
Triglycerides (<150 mg/dL)	97 ± 6	100 ± 6	0.92
LDL-C (<100 mg/dL)	103 ± 3	103 ± 3	0.91
LDL-p (<1000 nmol/L)	1038 ± 47	1005 ± 45	0.16
sLDL-p (<1317 nmol/L)	405 ± 38	365 ± 35	0.04^a^
HDL-C (<40 mg/dL)	58 ± 2	60 ± 2	0.95
HDL-p (24–49 umol/L)	35 ± 1	35 ± 1	0.90

^a^Lipoprotein Insulin Resistance Index [25]

### Changes in glucose-related parameters

Because α-CD treatment has previously been shown to improve glucose control in overweight patients [18], several tests related to glucose metabolism were also monitored (Table 8). Body weight, serum insulin, and HbA1C were unchanged by the α-CD treatment in the relatively healthy population examined in this study, but we did observe a small reduction in fasting glucose (−1.6 %; *p* = 0.05) in subjects when on α-CD versus placebo. The HOMA index did not show any difference but an insulin-resistance index based on NMR analysis [25] also showed a modest improvement (−11 %, *p* < 0.04) when subjects were on α-CD versus placebo. The mean BMI or ethnic distribution did not statistically differ between the responders with a reduction of the insulin-resistance index on α-CD and the non-responders, but the mean age of the responders (38 years) was significantly greater than the non-responders (31 years; *p* < 0.01).

**Table 8:** Changes in weight and glucose metabolism related parameters of subjects after 12–14 weeks on placebo or α-CD either measured by conventional biochemistry methods or by NMR (mean ± SEM)

Glucose metabolism	Placebo	α-CD	*p*
Weight (Kg)	74.8 ± 1.9	74.7 ± 2	0.99
Glucose (74–106 mg/dL)	88 ± 0.9	87 ± 0.7	0.05^a^
Insulin (2.6 − 24.9 mcU/mL)	7.1 ± 0.5	7.3 ± 0.6	0.56
Hgb A1C (4.0 − 6.0 %)	5.3 ± 0.4	5.3 ± 0.4	0.46
LIRI^a^ (%, by NMR)	1.6 ± 0.1	1.4 ± 0.1	0.04^a^
HOMA IR index	1.6 ± 0.1	1.5 ± 0.1	0.15

^a^Lipoprotein Insulin Resistance Index [25]

## Discussion

In the present study, we investigated the effect of α-CD supplementation on plasma lipid and glucose-related parameters in a relatively healthy population. In contrast, most of the other prior studies on α-CD were done on overweight patients or on patients with diabetes and or obesity [13, 18]. Although it was reasonably well tolerated and appeared to be safe, α-CD in this study only had minor beneficial effects on serum lipids and glucose, with unknown clinical significance.

The safety and tolerability of α-CD observed in this study is consistent with previous clinical trials [18, 19]. α-CD is largely non-absorbable [26] and when fully hydrolyzed produces glucose monomers and hence is thought to be relatively safe and has been granted GRAS status by the FDA. We did observe a non-significant increase in mild GI complaints when subjects were on α-CD compared to placebo, but 4 subjects did drop out of the study while on α − CD because of GI complaints. Although α-CD is non-absorbable, it can be hydrolyzed by gut bacteria [11, 26] and is thus considered a fermentable fiber, which could possibly account for the GI side effects.

In terms of lipid lowering, we observed a 10 % reduction in small LDL-particle number when subjects were on α-CD versus placebo, with no other changes in the lipid and lipoprotein profile. Small LDL has been shown in many studies to be particularly pro-atherogenic [22–24], presumably because of its ability to better infiltrate the vessel wall and it increased propensity for oxidation [27, 28]. Whether the change observed in this study is clinically significant in the absence of a total reduction in LDL-P or LDL-C is not clear. In previous animal studies, α-CD supplementation has been shown to reduce LDL-C [15], but the dose used in these studies was much higher than what was used in this clinical trial. In a prior clinical trial of α-CD on serum lipids, which used doses similar to our study, found a reduction in LDL-C of 11.9 ± 4.2 mg/dL after 3 months on the α-CD treatment, while the placebo group showed a 8.5 ± 6.2 mg/dL (*p* < 0.01) increase, but this only occurred in hypertriglyceridemic obese patients with BMIs > 30 kg/m^2^ [13]. In another small study of overweight individuals with a mean BMI of 26.9 kg/m^2^ and a mean age of 43.3 years, α-CD (6 grams per day) given for one month lowered LDL-C by approximately 6.7 % and a greater reduction was also seen in individuals with higher baseline triglycerides [18]. Because the subjects in the current study had a relatively normal lipid and lipoprotein profile at baseline, were younger (mean age of 34 years) than the previous studies and had a mean BMI of 25 kg/m^2^, this may have limited the effect from the α-CD treatment. Overall, these results suggest that α-CD may be more effective in lipid lowering in a more dyslipidemic and obese population, but this will have to be more definitively established in larger clinical trials.

Supplementation with α-CD was also found in the current study to cause a slight lowering of fasting plasma glucose and to improve the NMR-based insulin-resistance score [26]. Major changes, however, would not be expected in glucose metabolism based on the fact that we designed our selection criteria to recruit only healthy subjects and at baseline our subjects had a mean fasting glucose of only 86 ± 1 mg/dL, with a relatively low mean insulin of 6.6 ± 05 mcU/mL. In 2012, the European Food Safety Authority (EFSA) issued a scientific opinion approving the claim that the consumption of at least 5 g of α-CD in 50 g of starch will reduce post-prandial glycemic responses. The results from our study suggest, however, that at a dose of 6 g/day α-CD would have only a minimal effect in glucose control in non-obese healthy individuals.

The original rationale for why α-CD supplementation may lower serum lipids is that it would interfere with cholesterol or triglyceride absorption [13] like what has been described for other soluble fibers [5]. α-CD is known in vitro to bind to various lipids [15] but whether this process is relevant to fat absorption in vivo is not known [29]. Recently, it has been recognized that α-CD can be hydrolyzed and fermented by gut bacteria [30] and hence can be considered a pre-biotic. Supplementation with α-CD could, therefore, possibly change the composition of the gut flora in favorably ways that could improve lipid metabolism and insulin sensitivity [31, 32]. A similar mechanism has been proposed for how BAS and other soluble fibers may also improve lipid and glucose related parameters [5]. In a recent unpublished animal study in apoE-KO mice, we found by 16S ribosomal RNA sequencing that 1.5 % supplementation of α-CD markedly changed the gut microbiome, which has been shown modulate atherosclerosis [33, 34].

## Conclusion

In summary, α-CD supplementation was safe and reasonably well tolerated in a healthy population and had some minor beneficial effects in reducing small LDL-particle number and fasting glucose. Additional studies are needed to understand the consequence of such changes, the mechanism of action of α-CD and its effect on lipid lowering and glucose control in other patient populations.

## Abbreviations

BAS, bile acid sequestrants; HDL-C, high density lipoprotein cholesterol; LDL-C, low density lipoprotein cholesterol; LIRI, lipoprotein Insulin resistance index; TC, total cholesterol; TG, triglycerides; α-CD, α-cyclodextrin
