# Erratum to: Randomized double blind clinical trial on the effect of oral α-cyclodextrin on serum lipids

**DOI:** 10.1186/s12944-017-0435-4

**Published:** 2017-02-22

**Authors:** Marcelo J. A. Amar, Maryann Kaler, Amber B. Courville, Robert Shamburek, Maureen Sampson, Anna Wolska, Alan T. Remaley

**Affiliations:** Lipoprotein Metabolism Section, Cardio-Pulmonary Branch, National Heart, Lung, and Blood Institute, National Institutes of Health, Building 10, Room 8 N-228, 10 Center Drive MSC 1666, Bethesda, MD USA

## Erratum

After publication of the original article [1], it came to the first author’s attention that an additional contributor should have been included in the author list.

Dr Anna Wolska processed and analysed the NMR lipoprotein samples at the earliest stage of data analysis of this manuscript. Subsequently many other statistical analysis were performed and with the gap time between all the data to be analysed and the manuscript to be completed her important contribution was forgotten.

Dr Wolska has no competing interests to declare.
